# Effects of a Suspension Training Warm-Up on Maximal Cardiopulmonary Exercise Performance in Highly Active Adults: A Randomized Crossover Trial

**DOI:** 10.3390/jfmk11040385

**Published:** 2026-09-25

**Authors:** Robert J. Federico, Tiffany A. Caba-Abreu, Jamie Pigman, Tamara Rial Rebullido

**Affiliations:** Human Performance Lab, Department of Health and Physical Education, Monmouth University, West Long Branch, NJ 07764, USAjpigman@monmouth.edu (J.P.)

**Keywords:** young adults, maximal oxygen, peak oxygen uptake, warm-up exercise, suspension training

## Abstract

**Background and Objectives:** Warm-up before cardiopulmonary exercise testing (CPET) typically includes low-intensity aerobic activity. Whether a dynamic suspension-training warm-up represents a comparable alternative remains unknown. This study compared the cardiopulmonary responses between a treadmill walking warm-up (WW) and a suspension training warm-up (SW) before CPET in highly active young adults. **Methods:** Fifteen participants (6 females, 9 males; 21.2 ± 1.90 years) randomly completed both 6 min warm-up conditions in a randomized, counterbalanced order before an incremental treadmill CPET using a portable metabolic analyzer. Equivalence of peak oxygen uptake (VO_2_ peak) was assessed using two one-sided tests with a prespecified bound of ±1 MET (±3.5 mL/kg/min). **Results:** VO_2_ peak between SW (58.8 mL/kg/min; SD = 9.33) and WW (58.9 mL/kg/min; SD = 10.34) did not differ significantly (*p* = 0.957, d = −0.01). The 90% CI for the differences (−2.48 to 2.33 mL/kg/min) fell within the equivalence bounds (*p* = 0.010 and *p* = 0.013). Linear mixed model analyses revealed no significant effect of warm-up on VO_2_ peak (F_1,13_ = 0.003, *p* = 0.957) with no sequence-order effects (*p* > 0.05). Other peak CPET cardiopulmonary, perceptual, and performance outcomes did not differ between warm-ups (*p* > 0.05). **Conclusions:** A SW elicited equivalent VO_2_ peak and no significant differences in other CPET outcomes compared to a traditional WW in highly active young adults. These findings provide novel evidence that SW may represent a practical alternative warm-up before CPET in highly active young adults.

## 1. Introduction

Warm-ups are multifactorial, structured, and purposeful preparatory processes commonly performed before exercise to enhance cardiorespiratory, metabolic, and neuromuscular readiness for physical training and competition purposes. Warm-up protocols have been associated with a range of physical performance outcomes [1,2], although the effects vary depending on the type, intensity, and duration sequencing of the warm-up. Accordingly, the optimal structure and selection of warm-up activities may vary according to the demands of the subsequent activity, individual characteristics, training background, and contextual factors [1].

Warm-up protocols before cardiopulmonary exercise testing (CPET) traditionally consist of several minutes of low-intensity aerobic activity such as walking or cycling before a graded exercise test. Because CPET is designed to quantify maximal physiological capacity, a pre-test warm-up should adequately prepare the participant for maximal exertion without inducing fatigue or compromising the physiological outcomes being assessed. Importantly, the mode, intensity, and loading characteristics of a warm-up might influence cardiorespiratory responses before CPET [3]. Therefore, evaluating alternative warm-up modalities to determine whether they provide adequate preparation for CPET is warranted.

Suspension training represents a potential whole-body dynamic warm-up modality. Suspension training uses body weight as resistance through an adjustable strap system to perform a variety of dynamic whole-body, multi-joint exercises involving multiple muscle groups within a brief period. This type of whole-body preparation may increase cardiovascular and metabolic demand and provide a neuromuscular stimulus before subsequent exercise. A brief, dynamic whole-body warm-up may also alter physiological responses before exercise. However, the extent to which these acute changes influence subsequent maximal CPET responses remains unclear. Suspension training has been widely used in fitness and rehabilitation settings for its versatility, portability, and adaptability, as well as for its effects on core stability, balance, and neuromuscular control [4,5]. Moreover, suspension training sessions have been shown to elicit cardiometabolic responses consistent with aerobic exercise intensity guidelines [4]. However, the use of whole-body suspension training as a brief warm-up strategy to adequately prepare individuals for CPET without compromising cardiopulmonary responses remains unknown.

To the best of our knowledge, no studies have directly compared a dynamic whole-body suspension-training warm-up with a traditional aerobic warm-up before maximal CPET. Therefore, the purpose of this study was to compare cardiopulmonary responses during treadmill maximal CPET following a suspension-training warm-up (SW) and a continuous walking warm-up (WW) in highly active young adults. Primary outcomes included peak oxygen uptake (VO_2_ peak), peak heart rate (HR peak), and peak minute ventilation (V_E_ peak). Secondary measures included ventilatory outcomes, CPET termination time, and rate of perceived exertion (RPE). We hypothesized that a suspension-training warm-up would elicit equivalent VO_2_ peak and comparable physiological responses to a walking warm-up without compromising maximal performance.

## 2. Materials and Methods

### 2.1. Study Design

This study used a randomized, counterbalanced two-condition crossover study design. Participants were randomly assigned in a 1:1 ratio to one of two experimental sequences (SW-WW or WW-SW), before enrollment using Research Randomizer (www.randomizer.org, accessed on 5 October 2025). Simple randomization was used. The sequence was retained by the principal investigator (T.R.R). Participants and the rest of the researchers were not informed of the warm-up schedule until they arrived at the laboratory. Investigators were not blinded to condition. Sessions were performed on separate days with an interval of a minimum of 48 h to allow recovery from the previous CPET and a maximum of 7 days to limit changes in participants’ training or activity between sessions. Both sessions were conducted under standardized laboratory conditions and at the same time of day. Participants reported to the Human Performance Laboratory of Monmouth University (West Long Branch, NJ, USA). Participants were instructed to refrain from vigorous exercise for 48 h before testing, avoid alcohol for 24 h, and abstain from food for 3 h before testing. Participants were also instructed to wear the same shoes for both trial sessions. Participants were given standardized instructions before the start of the test and the same verbal encouragement during both testing procedures.

The reporting of this study follows CONSORT guidelines and their extension for crossover trials [6,7]. See Supplementary Table S1. This study was approved by the Institutional Review Board of Monmouth University (protocol code: SP2553 approved on 25 June 2025) and registered in ClinicalTrials.gov (Identifier: NCT07215052, registered in October 2025). The study protocol and planned statistical procedures are available in the prospectively published protocol before data collection [8]. There was no participant or public involvement in the design, conduct, analysis, or reporting of this study. No changes were made to the prespecified primary outcome after the trial started. Estimation of sample size was done with an a priori power analysis run by the software G-Power (version 3.1.9, Heinrich-Heine-University, Düsseldorf, Germany), with a desired power level of 0.80, an alpha level of 0.05, and a large effect size (Cohen’s d = 0.8) [9]. A total of 15 participants were calculated for a total power of 0.82.

### 2.2. Participants

Participants were highly active college-aged adults between 19 and 25 years of age who were classified as high activity on the International Physical Activity Questionnaire [10] and were able to participate in moderate to vigorous physical activity as determined by the Physical Activity Readiness Questionnaire [11]. Participants were excluded if they reported any cardiovascular, pulmonary, metabolic, neuromuscular, orthopedic, acute illness, or any other condition contraindicating maximal CPET or suspension exercise. Participant recruitment and experimental testing were conducted between November 2025 and May 2026.

A total of 16 participants were enrolled and randomized for the study (7 to the SW-WW sequence and 9 to the WW-SW). However, one participant assigned to the WW-SW sequence completed the first period but did not complete period 2 because of a scheduling conflict. The final sample comprised 15 participants (6 females, 9 males; 21.2 SD = 1.90 years). Participant characteristics are presented in Table 1.

### 2.3. Experimental Procedures

During each trial, participants rested in a seated position for 5 min before baseline vital signs were obtained. Baseline measurements included resting HR, blood pressure (BP), oxygen saturation (SpO_2_), body temperature, and RPE. Participants then completed the randomized warm-up condition (SW or WW), followed by a 2 min transition period between the warm-up and the CPET, during which vital signs were retaken. Following the CPET, participants completed a 1 min cool-down followed by 5 min of seated recovery, after which HR, BP, SpO_2_, body temperature, and RPE were reassessed. Figure 1 illustrates the experimental procedures and measurement time points.

The WW consisted of 6 min of treadmill walking at 3.0 mph with a 0% incline. The SW consisted of a 6 min dynamic exercise sequence performed using a Total Resistance Exercise training strap (TRX Training, Delray Beach, FL, USA), which included five different multi-joint upper-body and lower-body exercises with five 30 s rest intervals. These exercises were included as part of a practical whole-body suspension training sequence designed to engage multiple muscle groups in a brief six-minute routine. Participants performed each exercise at a comfortable, self-selected cadence while maintaining controlled movement and correct technique. No prespecified maximal number of repetitions was required. The suspension trainer was attached to the same fixed overhead anchor point for all participants. For the rows and push-ups, strap length was adjusted individually so that participants maintained a 45-degree angle relative to the floor. Both protocols were supervised by the research team members. The SW exercises, order, and duration are shown in Table 2. Before testing, participants received standardized instructions, practice, and demonstrated correct suspension training technique.

The treadmill CPET protocol was based on the proposed protocol by Green et al. [12], which is a valid and reproducible exercise protocol previously assessed in healthy adult populations. This protocol consists of 9 progressively increasing stages in treadmill speed and incline grade, with each stage lasting two minutes. The protocol starts at 5 mph (1.5% incline) and increases by 1 mph every two minutes, maintaining 1.5% incline during the first five stages. From stage 6 to 9, speed stays at 9 mph, and incline is increased from 1.5% to 10%, 12.5%, 15%, and 17.5% [12]. Age-predicted maximal heart rate (HR max) was calculated using the Gellish et al. [13] equation: HR max = 207 − (0.7 × age). This formula was selected because it is more accurate in the prediction of HR max in well-trained individuals [14]. Participants continued until volitional exhaustion. Predefined maximal criteria [15] were RPE between 9 and 10; attainment of HR max; or a steady state of VO_2_ despite increasing intensity. Total time of test completion was recorded. Monitoring of signs and symptoms requiring test termination was done throughout all sessions, and adverse symptoms were asked about during the trial and documented.

All anthropometric variables were measured following standardized procedures as described by the American College of Sports Medicine [15]. Height was measured using a portable stadiometer (seca 213, seca GmbH & Co, Hamburg, Germany) and body mass was measured using an electronic scale. Temperature was measured with an electronic thermometer (NC 200, Microlife AG, Widnaw, Switzerland). BP was assessed via an electronic device (M3, OMRON Healthcare Co., Ltd., Kyoto, Japan). The RPE was assessed using the Borg Category-Ratio 10 scale, ranging from 0 (nothing at all) to 10 (extremely strong/maximal) [16].

Expired gas exchange was measured breath-by-breath by using a calibrated, portable metabolic system (VO_2_ Master Pro Systems, Vernon, BC, Canada). The VO_2_ Master Pro system was calibrated before use with a one-point calibration that uses room air and a 3 L syringe. All metabolic data were collected via Bluetooth to the VO_2_ Master Pro mobile app (Vernon, BC, Canada) for storage and analysis. A Polar heart rate monitor and strap (Polar Electro Oy, Kempele, Finland) were integrated into the VO_2_ Master unit to record HR continually. The VO_2_ Master Pro has previously shown acceptable validity and test–retest reliability for ventilatory assessment [17,18]. VO_2_ Master Pro data were reintegrated to 30 s intervals for analysis and exported to Microsoft Excel.

### 2.4. Statistical Analysis

Descriptive statistics (mean ± SD) are reported for normally distributed variables and median and interquartile range (IQR) for non-normally distributed variables. Statistical analysis included only participants who completed both crossover conditions (*n* = 15). Equivalence testing of the two conditions was performed using two one-sided tests (TOST) with a priori bounds of ±1 MET (±3.5 mL/kg/min) for VO_2_ peak, as described in previous validation studies [19]. Equivalence was supported when both one-sided tests were statistically significant (*p* < 0.05), and the 90% confidence interval (CI) fell within the predefined equivalence bounds. Primary outcomes (VO_2_ peak, HR peak, and V_E_ peak) were analyzed using a linear mixed-effects model (LMM), with warm-up condition and sequence order as fixed effects and participant as a random intercept. Model assumptions were evaluated using residual Q-Q plots, residual versus predicted plots, and tests of residual normality. No substantial violations of assumptions were observed. Partial R^2^ was calculated for the fixed effects as a measure of effect size. Normality of paired differences was assessed using the Shapiro–Wilk test. If normality was violated, the Wilcoxon signed-rank test was used. Effect sizes are reported as Cohen’s d [20] for parametric comparisons and/or matched-pairs rank-biserial correlation for non-parametric comparisons with 95% CI. Exploratory LMM were additionally performed to examine whether baseline HR and baseline RPE were associated with termination time with warm-up condition and sequence order included as fixed effects and participant as a random intercept. Exploratory outcomes such as baseline, after warm-up, and recovery after CPET, physiological and RPE outcomes were analyzed using a 2 × 3 repeated-measures ANOVA. Significant interactions were assessed by Holm-adjusted pairwise comparisons, and sphericity was assessed using Mauchly’s test, with Greenhouse–Geisser corrections applied when the assumption of sphericity was violated. Partial eta squared was reported as the effect size (np^2^). Statistical analyses were conducted in Jamovi (version 2.3.21, Sydney, Australia) with the TOSTER module (version 0.4.0) and the GaMLj (version 2.8.0) modules. Statistical significance was set at *p* < 0.05.

## 3. Results

All fifteen participants completed the CPET, reaching volitional fatigue and satisfying at least one prespecified criterion for maximal effort. There were no adverse events. Measurements during baseline, post warm-up, and recovery after 5 min of CPET (HR, BP, SpO_2_, body temperature, and RPE) are presented in Table 3. Baseline and recovery vital signs and RPE were comparable between experimental conditions, indicating similar physiological status before and during post-CPET recovery. ANOVA repeated measures analysis revealed a significant condition × time interaction for HR (F (1.66, 23.26) = 5.85, *p* = 0.012; np^2^ = 0.295) where HR was significantly higher following SW than WW (mean difference: 16.87 bpm; *p* = 0.007). A significant condition × time interaction was also found for RPE immediately following the SW (F (2, 28) = 5.86, *p* = 0.007; np^2^ = 0.295), where RPE was also found to be significantly higher following the SW than WW (mean difference: 1.07, *p* = 0.036). No other significant condition × time interactions were observed for BP, body temperature, or SpO_2_ values.

Peak cardiopulmonary responses were similar between warm-up conditions (see Table 4). VO_2_ peak did not differ significantly between SW (58.84, SD = 9.33 mL/kg/min) vs. WW (58.91, SD = 10.34 mL/kg/min), and the effect size was trivial (*p* = 0.957, d = −0.01). Similarly, no statistical differences were observed for HR peak (SW: 196, SD = 6.52; WW: 195, SD = 5.01 bpm) or V_E_ peak. Equivalence testing bounds for VO_2_ peak were set at ±1 MET (±3.5 mL/kg/min) and indicated equivalence between warm-up conditions, where the 90% CI (−2.48 to 2.33 mL/kg/min) fell within the predefined equivalence bounds. Both one-sided tests were statistically significant (TOST lower bound: *p* = 0.013 and TOST upper bound: *p* = 0.010), supporting equivalence between conditions. The LMM analyses also revealed no significant effect of warm-up condition on VO_2_ peak (F_1,13_ = 0.003, *p* = 0.957, partial R^2^ < 0.001) or HR peak (F_1,13_ = 0.637, *p* = 0.439, partial R^2^ = 0.047) or V_E_ peak (F_1,13_ = 0.139, *p* = 0.715, partial R^2^ = 0.011). Moreover, there were also no significant sequence-order effects observed for VO_2_ peak (*p* = 0.462), V_E_ peak (*p* = 0.765), or HR peak (*p* = 0.051), although the HR peak sequence effect was close to the conventional threshold for statistical significance. No significant between-condition differences were observed for the secondary peak outcomes O_2_ pulse, respiratory frequency, tidal volume, or peak RPE (*p* > 0.05). Moreover, no significant differences were found for CPET termination time between conditions (SW: median 17.1 min; IQR = 2.44; WW: median 17.0 min, IQR = 1.84; Wilcoxon W= 48.0, p = 0.514). In secondary exploratory LMM analyses, baseline HR was found to not be associated with CPET termination time (estimate = 0.009 min per bpm, 95% CI −0.018 to 0.037, *p* = 0.502). However, baseline RPE showed a borderline association with CPET termination time (estimate = 0.50 min per unit increase, 95% CI = 0.01 to 0.99, *p* = 0.050).

## 4. Discussion

The present study examined the cardiopulmonary effects of a traditional treadmill low-intensity aerobic warm-up compared to a dynamic warm-up performed with dynamic suspension training exercises in highly active young adults. Our initial hypothesis that an SW would be a comparable warm-up to a WW preceding maximal CPET was supported for the primary equivalence outcome. Specifically, VO_2_ peak was equivalent between warm-up conditions and no significant between-condition differences were observed for the other peak cardiopulmonary outcomes. Additionally, HR and RPE were higher immediately after SW, indicating greater acute physiological and perceptual demand before CPET. These findings indicate that a brief, dynamic SW may provide a practical alternative to a traditional treadmill warm-up before CPET in highly active young adults, although its greater acute physiological and perceptual demand should be considered.

Our findings are consistent with previous research suggesting that variations in warm-up protocols might have limited influence on maximal cardiopulmonary responses. Recently, Pedrosa et al. (2025) reported that changing the intensity and duration of aerobic warm-ups did not significantly alter VO_2_ peak in healthy young adults following maximal effort CPET cycle ergometry [21]. Our findings extend those of Pedrosa et al. [21] by suggesting that providing flexibility in the mode of warm-up may be possible without compromising maximal cardiopulmonary outcomes. Similarly, Faigenbaum et al. [22] reported no significant differences in maximal aerobic exercise performance following an aerobic walking warm-up compared to a 6 min dynamic bodyweight protocol in youth. In a follow-up study, Faigenbaum et al. (2024) tested a dynamic medicine ball warm-up with an aerobic warm-up in youth and reported comparable VO_2_ peak values, although some submaximal cardiopulmonary differences were noted [23]. Collectively, these findings support the notion that different warm-up strategies based on dynamic bodyweight exercises may provide sufficient physiological preparation for maximal CPET without altering peak outcomes. Our findings extend emerging evidence to highly active adults and represent the first study using a suspension exercise modality as a warm-up alternative to aerobic low-intensity warm-ups.

Our study found higher HR and RPE after the SW. This finding is consistent with Faigenbaum et al. (2024), who found that a dynamic warm-up elicited higher HR before CPET than a treadmill walking warm-up in youth [23]. Peak RPE during CPET did not differ significantly between conditions. Although HR and RPE were higher immediately after SW, these acute physiological and perceptual demands were not accompanied by differences in CPET termination time or peak perceived exertion. Thus, the immediate physiological and perceptual demand of the SW did not compromise subsequent maximal CPET performance in this highly active population. 

Although both warm-ups were matched in duration, they were not matched for exercise intensity or workload. The neuromuscular demand of the SW exercises, which included lower-body movements such as squats, reverse lunges, and squat jumps, performed with only brief rest intervals, might have contributed to the greater immediate physiological and perceptual demand. However, neuromuscular and metabolic markers were not assessed to confirm these potential mechanisms. Of interest, baseline RPE and CPET termination time showed a borderline positive association, suggesting that pre-exercise perceptual state may be related to exercise duration. However, this should be interpreted cautiously given the exploratory nature of the analysis and small sample size. 

The present findings have practical applications for the sport and exercise science community and for exercise testing in active adults. Expert-based recommendations for warm-up recognize that warm-up strategies should be context-dependent and consider the demands of the subsequent activity as well as individual characteristics instead of following one single warm-up approach [1]. The comparable cardiopulmonary responses observed in this study suggest providing practitioners flexibility in warm-up modality selection for maximal aerobic exercise performance. A greater flexibility choice might allow practitioners to consider individual preferences, training background, perceptual readiness, equipment options, and other variables when selecting a warm-up modality before CPET. However, given the higher perceptual and physiological demands right after the SW, practitioners should consider the greater acute demand of this dynamic warm-up when selecting a strategy before CPET.

Several study strengths should be acknowledged. The randomized, counterbalanced crossover design was selected so each participant could serve as their own control and reduced between-participant variability. The use of equivalence testing with a prespecified bound strengthened the interpretation of the primary study outcome. Nonetheless, some limitations should be considered. Firstly, although the sample size was calculated and the within-subject design increased experimental control, the relatively small sample size was powered to detect a large between-condition effect rather than for equivalence analysis using a prespecified margin. Therefore, the VO_2_ peak equivalence findings should be interpreted with this limitation in mind. Secondly, the sample consisted exclusively of young, highly active participants, which limits generalization of the results to other populations such as older populations, less active or clinical populations. Participants’ high activity levels and reported prior familiarity with resistance and bodyweight exercise may also have attenuated the physiological and perceptual responses to the SW compared with those that might be observed in less trained populations. Further studies should replicate these findings in more diverse populations. Thirdly, although body temperature did not differ significantly between conditions, the small decrease observed after both warm-ups may reflect measurement variability associated with the use of a peripheral electronic thermometer. Lastly, only one protocol of a fixed duration was assessed. Although exercise duration and sequence were standardized, cadence was self-selected, and repetitions were not recorded. Therefore, the external workload of the SW was not quantified and may have varied among participants. Different warm-up durations, intensities, or exercises were not assessed. Future studies should also investigate different suspension training doses and exercise configurations as well as assess responses across different populations and fitness levels.

## 5. Conclusions

This study contributes to an evolving body of research examining warm-up strategies preceding CPET and assessing their differential outcomes. Our findings demonstrated that a brief SW elicited equivalent VO_2_ peak and no significant differences in other peak cardiopulmonary outcomes compared to a traditional WW in highly active young adults. Although SW elicited greater acute physiological and perceptual demand immediately after the warm-up, these differences did not impair maximal CPET performance. These findings provide novel evidence that suspension training could represent a practical warm-up strategy in highly active adults.

## Figures and Tables

**Figure 1:**
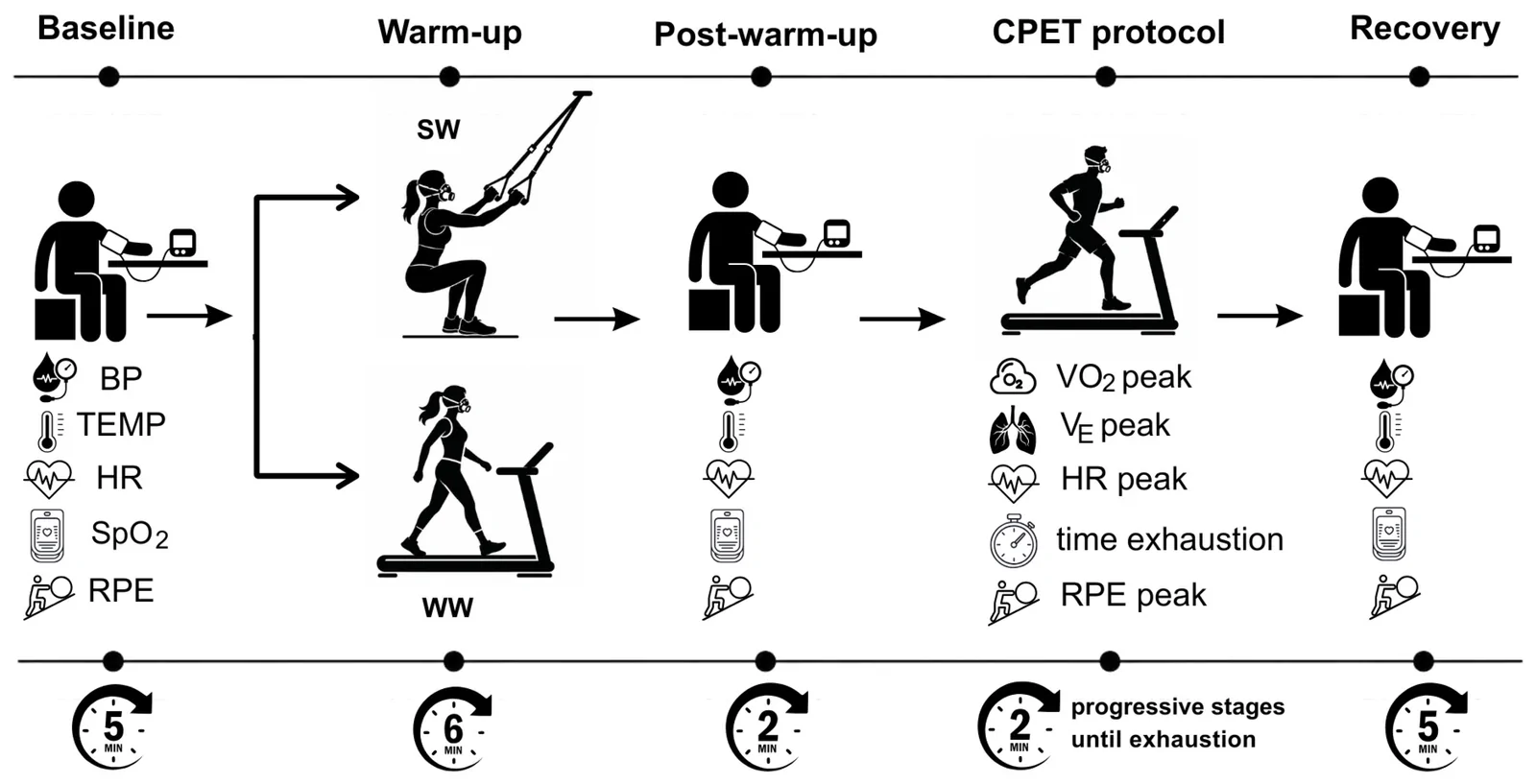
Schematic figure of the experimental procedure.

**Table 1:** Descriptive characteristics of the participants.

Characteristics	Total Sample (*n* = 15)	Males (*n* = 9)	Females (*n* = 6)
Age (years)	21.2 (1.90)	21.4 (2.19)	20.8 (1.47)
Body mass (kg)	74.0 (15.9)	84.3 (8.42)	58.5 (10.7)
Height (cm)	173 (13.7)	181 (7.87)	161 (11.6)
BMI (kg/m^2^)	24.3 (3.02)	25.7 (2.34)	22.2 (2.85)

**Table 2:** Dynamic warm-up exercises with suspension training straps.

Order	Warm-Up Exercise	Duration (Seconds) *
1	Reverse Lunges	45
2	Squats	60
3	Jump Squats	45
4	45-degree rows	30
5	45-degree push-ups	30

* 30 s rest intervals.

**Table 3:** Descriptive physiological values and RPE during baseline, immediately after warm-up condition, and 5 min post-CPET recovery.

Variable	Baseline SW	Baseline WW	Post SW	Post WW	Recovery SW	Recovery WW
Heart Rate (bpm)	77.7 (10.2)	74.4 (12.6)	113.0 (19.3) *	95.9 (18.0)	115.0 (12.0)	111.0 (10.1)
Systolic BP (mmHg)	122 (15.8)	121 (17.3)	131.0 (6.40)	128 (11.4)	121 (11.0)	123 (12.9)
Diastolic BP (mmHg)	72.5 (11.0)	72.1 (7.80)	78.4 (10.0)	80.4 (8.3)	74.5 (10.9)	73.5 (11.2)
SpO_2_ (%)	97.3 (1.87)	98.2 (0.86)	97.7 (1.10)	98.5 (0.74)	95.1 (1.53)	95.7 (1.72)
Temperature (°F)	98.3 (0.76)	98.2 (0.93)	97.3 (0.80)	98.1 (0.81)	97.7 (1.07)	97.7 (0.83)
RPE (0–10)	0.71 (0.82)	0.60 (0.73)	2.33 (0.97) *	1.27 (0.88)	2.20 (1.08)	2.27 (1.02)

Values are presented as mean (SD). * Significant differences between warm-up conditions at the corresponding time point; Holm-adjusted pairwise comparisons *p* < 0.05.

**Table 4:** Peak cardiopulmonary and performance outcomes during CPET following SW versus WW.

Variable	SW	WW	Difference (SW-WW) (95% CI)	*p*-Value	Effect Size
HR peak (bpm)	196 (6.52)	195 (5.01)	0.79 (−1.34, 2.91)	0.439	0.21
VO_2_ peak (ml/kg/min)	58.84 (9.33)	58.91 (10.34)	−0.08 (−3.01, 2.86)	0.957	−0.01
Absolute VO_2_ peak (L/min)	4.433 (1.228)	4.445 (1.279)	−0.011 (−0.242, 0.219)	0.918	−0.03
V_E_ peak (L/min)	123.98 (28.74)	124.86 (27.44)	−0.88 (−6.00, 4.23)	0.715	−0.10
O_2_ pulse peak (mL/beat)	22.70 (6.32)	22.86 (6.69)	−0.16 (−1.24, 0.92)	0.756	−0.08
Respiratory frequency peak (breaths/min)	51.24 (6.22)	52.57 (5.39)	−1.33 (−3.53, 0.88)	0.215	−0.35
Tidal volume peak (L)	2.64 (0.67)	2.59 (0.59)	0.05 (−0.08, 0.18)	0.402	0.23
Test termination time (min) †	17.1 (2.44)	17.0 (1.84)	--	0.514	−0.200
Peak RPE (0–10) †	10.0 (2.00)	10.0 (1.00)	--	0.222	−0.500

Values are presented as means (SD) for normally distributed outcomes, except for the non-normally distributed outcomes of termination time and peak RPE, which are presented as median (IQR); between-condition differences are presented as mean differences with 95% CIs and effect sizes as Cohen’s d values for normally distributed outcomes, obtained from paired-samples *t*-tests. For outcomes with non-normally distributed paired differences (test termination time and peak RPE), † Wilcoxon signed-rank tests and matched-pairs rank-biserial correlations were reported.

## Data Availability

De-identified data and the data dictionary are available from the corresponding author upon request. The statistical output is also available by the corresponding author by request.

## Supplementary Materials

The following supporting information can be downloaded at: https://www.mdpi.com/article/10.3390/jfmk11040385/s1, Table S1: CONSORT 2025 checklist item description adapted from the CONSORT 2010 extension to randomized crossover trials.

## Abbreviations

The following abbreviations are used in this manuscript:

BMI	Body mass index
BP	Blood Pressure
CI	Confidence intervals
CPET	Cardiopulmonary exercise testing
ES	Effect size
LMM	Linear mixed-effects methods
HR	Heart Rate
HR peak	Peak heart rate
HR max	Maximal heart rate
RPE	Rate of perceived exertion
SD	Standard deviation
SW	Suspension training warm-up
TOST	Two-one-sided tests
VO2 peak	Peak oxygen uptake
VE	Expired ventilation
WW	Walking warm-up
